# Selectivity and random-access in automatic analysers

**DOI:** 10.1155/S1463924688000343

**Published:** 1988

**Authors:** Pierangelo Bonini, Ferruccio Ceriotti, Carlo Franzini

**Affiliations:** 1 Istituto Scientifico San Raffaele Via Olgettina 60 Milano 20132 Italy; 2 Ospedale di Rho Corso Europa 250 Milano 20017 Rho Italy


Journal of Automatic Chemistry Vol. 10, No. 4 (October–December 1988), pp. 167–170

### Selectivity and random-access in automatic analysers

**Pierangelo Bonini, Ferruccio Ceriotti**
*Istituto Scientifico San Raffaele, Via Olgettina 60, 20132 Milano, Italy*

**and Carlo Franzini**
*Ospedale di Rho, Corso Europa 250, 20017 Rho, Milano, Italy*

*Progress in automation in clinical chemistry has featured in recent years a change from rigid to very flexible instruments. The latest instruments are able to perform on each sample only those tests requested, and often do so in a random sequence. The technological aspects of this progress in instrument design are described in this paper (these include computerization of analytical steps, multiple wavelength technology, dry chemistry systems, new designs of centrifugal analysers and capsule chemistry). Some problems with terminology (for example selective, random and batch-selective analysers) are discussed.*

The first step in illustrating the concept of random analysers should be a definition of the term 'random'. There is actually some uncertainty here: some authors, for example, have suggested replacing the word 'random' with 'selective' [1]. Therefore, rather than starting with terminology, it is better to first illustrate how automation in clinical chemistry has evolved, in the last 10–15 years, from very rigid to flexible instruments [2]. Problems with terminology will then be discussed taking into consideration the most relevant technical features of the instruments which are currently available.

### Non-selective analysers

Figure 1 presents a general model of a discrete, sequential automatic analyser, represented by a light source, a filter carousel or other monochromatic device and a series of cuvettes with reaction mixtures of the same colour (and obviously different intensity), a detector and an analogue-digital converter.

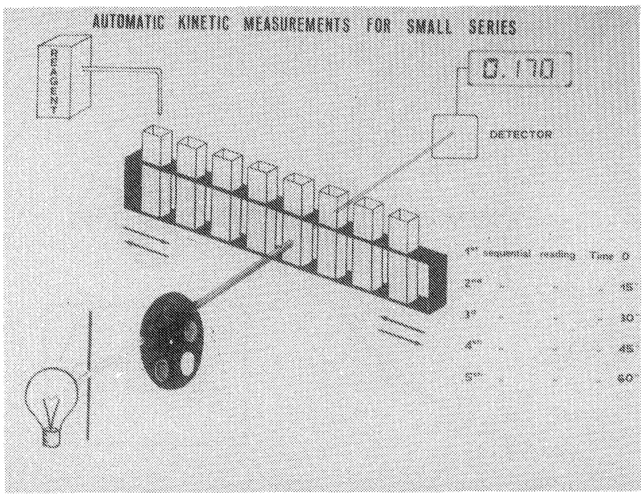

*Figure 1. Schematic diagram of a discrete, sequential automatic analyser.*

This system, working at a wavelength fixed for each analytical batch, represents a traditional solution, which is very appropriate for automatic kinetic measurements in reasonably sized batches or, in a slightly modified version, for automatic measurements of large end-point reaction batches.

Obviously, in this kind of instrument, samples must be preselected according to the tests required (for example, all the samples for glucose testing first, then all the samples for urea and so on). This requires a great deal of work to be carried out manually for the preparation of samples, and carries a high risk of sample mismatching. This kind of instrument is typically an inflexible analyser! If two or more analytical units similar to this are assembled a multichannel instrument is obtained.

Multichannel analysers, at least in the most recent models, have a certain degree of flexibility, as the operator can select the tests requested on each specimen. These instruments, despite their great success in the 1970s, especially in large laboratories which have very large numbers of test requests, have some disadvantages: they are bulky instruments, as well as being complex and expensive.

In these instruments, the throughput, that is the number of tests performed per unit of time, is correlated to the number of tests requested on each sample, the number of samples processed per unit of time being absolutely fixed.

The great success of the so-called biochemical profiles in the 1970s depends, at least in part, on this consumistic approach to automation: the need to justify big analysers in some cases brought, as a consequence, the adoption of non-selective profiles in clinical and other laboratories.

### The concept of flexibility in automation

What clinical laboratories actually need are simple instruments, possibly with only one analytical channel which can perform any requested test in any sequence on any specimen. In an ideal instrument it should be possible to load samples in a random and continuous way and get the results, patient by patient, in the shortest time possible.

This is what has been obtained with the last generation of instruments. The following examines how technological progress has influenced the design of automatic analysers.

Advances in electronics and microprocessor technology have, of course, played a central role.

Let us consider how many functions, in a modern automatic analyser, can be computer controlled (see figure 2). Starting from the top part of figure 2 we see the identification of the sample, represented by a camera, then on the right, the control of fluid volumes, represented by a syringe, time and temperature control, selection of appropriate wavelength, represented by a filter carousel and by a combination of a mirror-grating and a multiple photodetector system, calculations, control of kinetic reactions and, finally automatic trouble shooting.

All of these functions are computer controlled in a modern analyser, which substantially improves the instrument's flexibility.

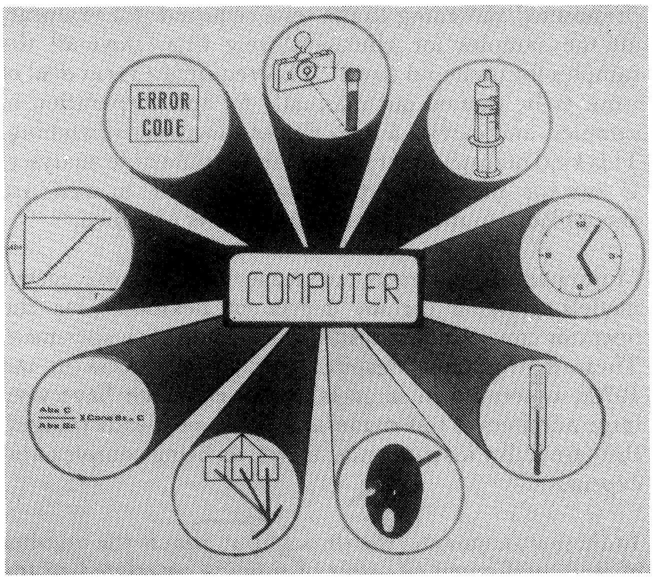

*Figure 2. Computer-controlled functions.*

### Selective–random analysers

The following illustrates how some instrumental and analytical advances have influenced the flexibility of instruments. Figure 3 shows a multiple wavelength system: a polychromatic light passes through the reaction tube, then it is reflected and dispersed by a mirror-grating; various photodetectors are then simultaneously activated by the reflected light beams at different wavelengths. This is possible because of the progress in the field of monochromators, with the introduction of mirror-gratings; the signals recorded by the different photodetectors are then processed by a computer. In this way, different colours, in sequential tubes, can be easily read and different reactions can be performed and read in sequence on the same reaction mixture, by dispensing additional reagents.

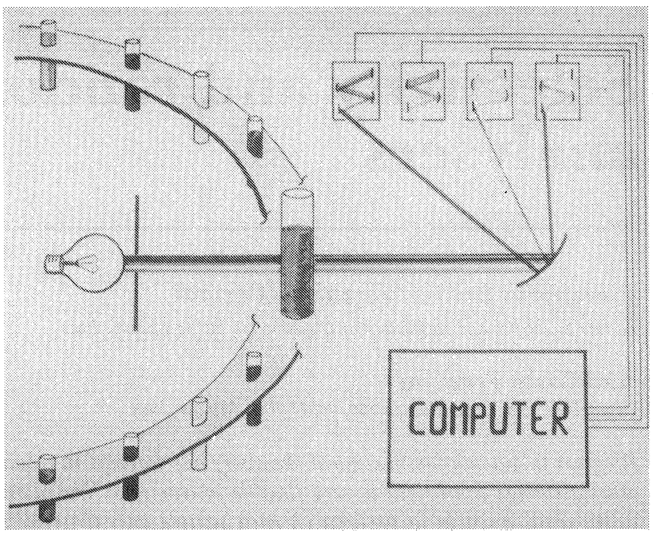

*Figure 3. Schematic diagram of a selective-random analyser.*

In this way the problem of random loading of samples is solved; as is the problem of sample blank subtraction and the flagging of high interference samples, through the polychromatic technique. A disadvantage of these instruments is a comparatively limited throughput; to increase it, the design of these instruments has been modified so that more than one sample can pass through a single analytical step (for example pipetting and reading) simultaneously. A slight modification of this technology is illustrated in the figure 4 – the system of multiple photodetectors is replaced by a filter carousel selecting the wavelength after the light has crossed the reaction tube.

Technical arrangements for random monitoring of different analytical reactions in subsequent tubes, or even in the same tube, lead to a great improvement in instrument flexibility. The same result can be obtained through the adoption of a television camera tube (for example Vidicon) or another type of array detectors which can scan the whole optical spectrum. This approach has not

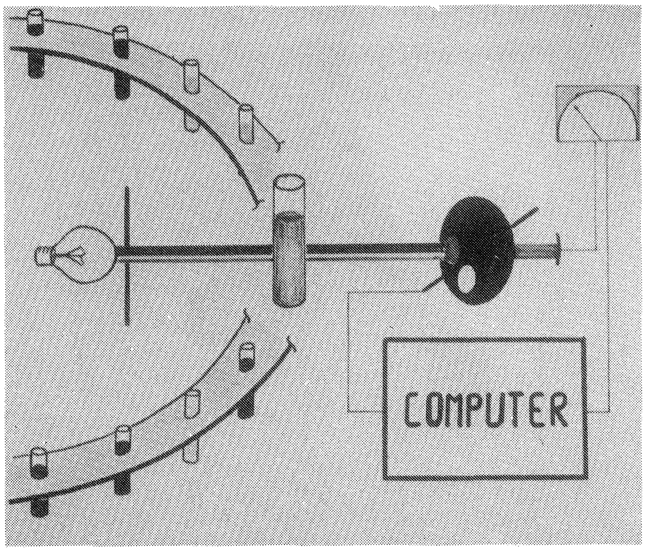

*Figure 4. Schematic diagram of a selective-random analyser.*

been very successful so far, possibly because of high costs, low sensitivity, the need to find a compromise between spectral resolution and spectral range and possible photochemical interference of the undispersed energy with a photosensitive molecule.

Reagent carry-over can be a problem in some of the random-access analysers which use liquid reagent [3]; this does not happen with instruments that use dry chemistries (see figure 5).

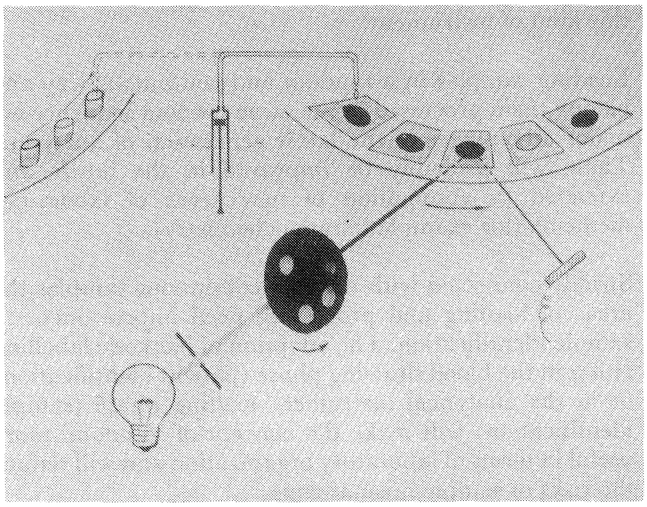

*Figure 5. Schematic diagram of a selective-random analyser using dry reagents.*

The cost of reagents is usually higher in dry chemistry, although there are many advantages: for instance better standardization, elimination of preparation work on reagents, less reagent waste, and, in many cases, an excellent analytical quality.

Centrifugal analysers, which were typically non-selective in the first models, have also been modified in recent years. Figure 6 shows how a beam of light after crossing the reaction tube is reflected toward a rotating filter carousel; in this way all the tubes containing different reaction mixtures are scanned at all the wavelengths available, this results in the possibility of performing any kind of test on any specimen either at the same time or in very close sequence.

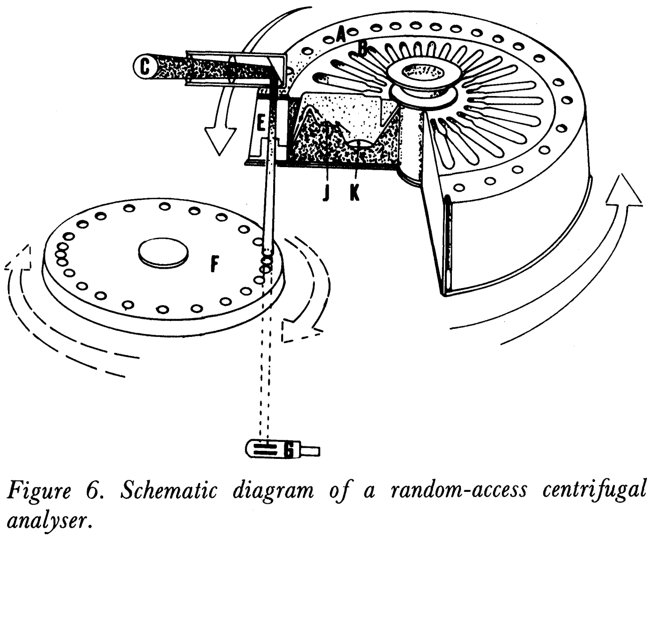

*Figure 6. Schematic diagram of a random-access centrifugal analyser.*

In most selective instruments illustrated here, it is a rule that the longer time of monitoring a reaction, the lower the throughput of the instrument. To overcome this drawback, two alternative approaches have been adopted: some companies have assembled many analytical channels which operate simultaneously, while the other alternative is represented by another innovative technology, which is based on the rotation of the light source (instead of reaction tubes). In this kind of instrument (figure 7) a relatively high throughput can be obtained, with a longer monitoring time than any of the previously described technologies [4].

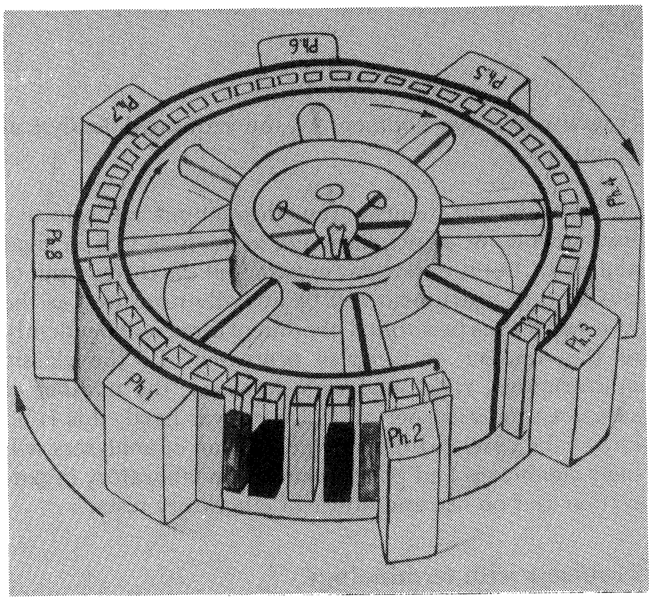

*Figure 7. Schematic diagram of a random-access analyser with rotating light source.*

There was a general feeling until about three years ago, that the concept of random analysis could never be applied to continuous flow automation. However, an innovative technology, called capsule chemistry, was presented at about that time. Random analysis (i.e. any analyte on any sample in any sequence) was shown to be very successfully performed on a continuous flow analyser. A capsule is a single reagents-sample unit (see figure 8), moving in a continuous flow tube and separated by the next and by the previous capsules by a special inert fluid, which eliminates any risk of sample or reagent carryover. Whilst the capsule moves in a certain area (see the left-hand side of figure 8), with a small diameter tube, there is no possibility of the serum mixing with reagents, because they are separated by a small air bubble, but, as the capsule reaches the so-called 'vanishing zone' (indicated in figure 8 as number 2) the diameter of the tube becomes larger, with consequent mixing of serum and

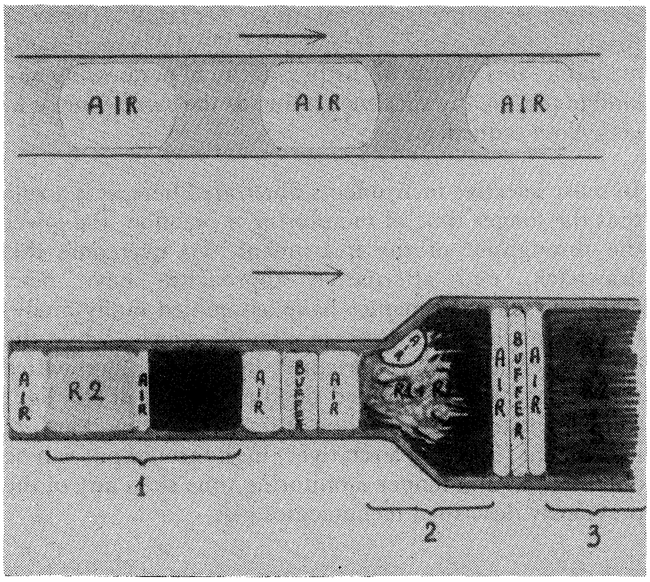

*Figure 8. Schematic representation of continuous flow and 'capsule flow'.*

reagents and devleopment of the requested analytical reaction [5].

As already mentioned, the throughput of selective instruments, in principle, is not as high as it is in rigid analysers, but is important to note that the throughput of this kind of instrument can be actually measured as the number of tests performed per unit of time, independently of the number of samples processed. In the classic multichannel, rigid analysers, however, the number of samples processed per unit of time was fixed; this is no longer the case and the new generation of analysers will have significant and positive effects on laboratory organization and on use and abuse of laboratory tests.

### Problems with terminology

Problems exist with the terms 'selectivity' and 'random'. 'Selective' could be defined as an analyser where only the tests requested on each sample are performed, and 'random' as an analyser where any analyte can be determined in any sequence on any specimen, which implies the elimination of any carry-over. All random analysers are, of course, selective; for many instruments 'selectivity' and 'random' could be considered as synonymous, but this is not the case for those instruments where samples, loaded in a random way, are then processed in a batch sequence (that is test by test, rather than sample by sample).

In these instruments, sample loading must be completed before the analytical phase starts and no patient report is available before the analytical phase is completed, which represents a major constraint on laboratory organization. The term 'batch-selective' has been suggested to indicate this kind of instrument.

Loading samples in a random and continuous way and having them processed in the same random sequence are major advantages of the latest generation of analysers. These will probably be improved in the future and extended to automation of new areas of laboratory medicine (for example immunochemistry).

Strictly connected with random-continuous samples the areas of loading and processing need improvement in sample identification. The adoption of bar code labelling either in the blood drawing phase (patient identification) or in the analytical instrument loading phase (sample identification) will make the concept of 'random' more useful in terms of laboratory organization and will reduce the risks of sample mismatching.

### Acknowledgement

This work was partially supported by CNR (Consiglio Nazionale delle Ricerche).

### References

1. Haeckel, R., Document of Analytical Chemistry and Clinical Chemistry Division of IUPAC (International Union of Pure and Applied Chemistry) (in press).
2. Bonini, P. A., *Pure and Applied Chemistry*, **54** (1982), 2017.
3. Dell'Anna, C., Morosini, L., Franceschin, A. and Bortolussi, A., *Clinical Chemistry*, **32** (1986), 1411.
4. Mitchell, F. L., in *Centrifugal Analysers in Clinical Chemistry*, Eds. C. P. Price and K. Spencer (Praeger, 1980), p. 311.
5. Cassaday, M., Diebler, H., Herron, R. *et al.*, *Clinical Chemistry*, **31** (1985), 1453.

